# Increasing antibody yield and modulating final product quality using the Freedom^TM^ CHO-S^TM^ production platform

**DOI:** 10.1186/1753-6561-5-S8-P102

**Published:** 2011-11-22

**Authors:** Michelle Sabourin, Ying Huang, Prasad Dhulipala, Shannon Beatty, Jian Liu, Peter Slade, Shawn Barrett, Shue-Yuan Wang, Karsten Winkler, Susanne Seitz, Thomas Rose, Volker Sandig, Peggy Lio, Steve Gorfien, Laurie Donahue-Hjelle, Graziella Piras

**Affiliations:** 1 Life TechnologiesTM, Frederick, MD, 21704, USA; 2 Life TechnologiesTM, Eugene, OR, 97402, USA; 3 Life TechnologiesTM, Grand Island, NY, 14072, USA; 4 ProBioGen AG, Goethestrasse 54, 13086 Berlin, Germany

## Background

Cell line development (CLD) is a critical step in the generation of biotherapeutics, but it is still hindered by several pain points, including the lengthy and labor-intensive workflow needed to isolate desirable clones, lack of reproducibility, as well as potential protein quality issues. Over the last decade, antibody titers in mammalian cell culture systems in excess of 3 g/L have been achieved through the use of novel media and feeds. However, it is still a challenge to consistently and rapidly create a stable cell line and a cell culture process capable of supporting both high antibody yield and acceptable post-translational modifications while managing the effort required for execution of the workflow. The goal of the study was to develop a robust and reproducible stable cell line workflow to generate scalable high-producing clones in less than 6 months, with industry-standard titers and desirable product quality using minimal effort.

Using CHO-S™ as the host cell line, we first evaluated if a single medium could be used for the entire CLD workflow, therefore avoiding the issues and complications of changing media during this process. We investigated if a formulation previously shown to increase titer as a production medium could in fact be used for all CLD steps, from transfection to stable pool isolation all the way through to clone productivity, without compromising titers or performance. The same rich production medium was used in limiting dilution cloning and compared to a lean cloning medium prototype. Furthermore, robustness of the workflow was verified by testing multiple molecules. We also explored reducing effort by streamlining all the steps of the workflow. Finally, we assessed top clone scalability and expressed product quality. We tested whether clones chosen only by titers responded well to scale-up and process development in a model bioreactor setting. In addition, product glycosylation from these clones was compared to the same molecule produced in CHO DG44 cells, a well-characterized production platform.

## Results

**Figure 1:**
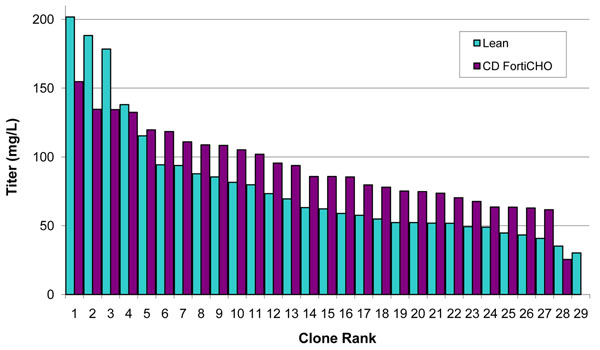
Cloning medium choice does not impact clone titer distribution. The same Molecule 1-producing stable pools were seeded for limiting dilution cloning in either a lean cloning medium prototype or CD FortiCHO^TM^ Medium. Following clone scale-up, the top clones from each process were assessed in shake flasks using a simple fed-batch process; day 7 data are shown. While there are differences in the absolute numbers, especially for the top 3 clones, overall the clones produced similarly whether they were isolated from lean cloning medium prototype or from CD FortiCHO^TM^ Medium.

Our results show that cell growth during selection and productivity assessment were affected by both the basal medium and nutrient feed strategy. Stable pools generated in either CD OptiCHO^TM^ Medium or CD FortiCHO^TM^ Medium gave the most desirable outcomes in terms of recovery time and productivity. We also found that CD FortiCHO^TM^ Medium can be used for every step of the CLD workflow, including limiting dilution cloning. When we performed a limiting dilution cloning using the same pool seeded in either a lean cloning medium prototype or in CD FortiCHO^TM^ Medium, we found that the top 10 clones isolated from each medium showed similar average titer trends (Figure 1). We also established the robustness of the selection scheme by demonstrating that all tested molecules show an increase in cell pool titer during a two-stage selection scheme that uses simultaneous puromycin and methotrexate treatment. In addition, the resulting clones can readily be scaled up to bioreactors: clones that were producing ~0.5 g/L in simple fed-batch (glucose feed only) using shake flasks readily scaled-up to a DasGip bioreactor and produced up to 3.2 g/L under fed-batch mode with minimal process development (Table 1). Finally, we demonstrated that the majority of clones isolated are stable for productivity, and that the glycosylation pattern of the same molecule produced in either CHO DG44 or CHO-S^TM^ cells was indistinguishable.

**Table 1:** Molecule 1 clone productivity during scale-up from shake flask to bioreactor.

Molecule 1 clone	Shake flask simple fed-batch (g/L)	Shake flask fed-batch (g/L)	Bioreactor fed-batch (g/L)
1	0.59	0.87	2.2
2	0.55	1.06	1.5
3	0.52	0.96	3.3

The top 3 clones expressing Molecule 1 were cultured in CD FortiCHO^TM^ Medium in shake flasks using a simple fed-batch or fed-batch process, and in a DasGip bioreactor using a fed-batch process. Clone titers increased 3- to 6-fold from simple fed-batch shake flask culture to fed-batch bioreactor culture.

## Conclusions

The use of a single medium for the entire workflow is revolutionary, and has the distinct advantage of avoiding any need for media adaptation at any point in the workflow, whether it be preceding or following cloning, or for productivity assessment. This in turn avoids any undesirable genetic selection that may occur during such adaptation steps, and facilitates streamlining the workflow for ease of use and efficiency. Clones are easily scaled from shake flask to bioreactor and produce 2-3 g/L with minimal process development. Product glycosylation in top CHO-S^TM^ clones was comparable to historical data from CHO DG44-derived clones expressing the same molecule. In addition, the establishment of clone stability and acceptable glycosylation patterns are key attributes required for regulatory approval of biotherapeutic production. Together, these results demonstrate the capabilities of the Freedom^TM^ CHO-S^TM^ Kit as an efficient and robust stable CLD platform, which can be accessed without the burden of milestone or royalty payments.

